# About the Degeneration of a Mediastinal Bronchogenic Cyst

**DOI:** 10.5146/tjpath.2020.01492

**Published:** 2021-05-15

**Authors:** Mona Mlika, Rahma Ayadi, Faouzi Mezni

**Affiliations:** Abderrahman Mami Hospital, Department of Pathology, Ariana, Tunis

**Dear Editor,**

Mediastinal cysts are benign lesions of mediastinum whose accurate diagnosis is based on the microscopic analysis of the lining epithelium and the cyst wall (1,2). The degeneration of mediastinal cysts has been rarely reported in the literature. The authors aimed to describe a well illustrated case of degenerated mediastinal cyst.

The authors report the case of a 31-year-old man, without a particular past medical history but with a smoking habit. The patient presented with a cough associated with dyspnea, and a weight loss of 12 kg in a 3-month period. Physical examination revealed a blood pressure of 130/80, a heart rate of 74 bpm, a temperature of 37oC, and a respiratory rate of 16 cycles/min. A search for the Koch bacillus in the sputum was negative. Chest-X-ray revealed a latero-tracheal shadow measuring 10 cm that was situated in the posterior mediastinum (Figure 1). The CT-scan revealed a posterior and mediastinal mass in contact with D2 and D4 measuring 82x68 mm, without a costal invasion or lymph node metastases (Figure 1). The diagnoses of a neurogenic tumour or a bronchogenic cyst were suspected. Cystectomy was performed. Perforation of the cyst wall was reported. The gross examination revealed a cystic mass with a necrotic content measuring 80x60x10 mm (Figure 2). The microscopic exam revealed a cystic wall with a largely ulcerated lining and preservation of foci lined by a single cell lining with some calcifications (Figure 3). Tumour proliferation was observed within the cystic fibrous wall. The epithelium lining was ciliated (Figure 3). The carcinomatous proliferation was solid and papillary. Tumour cells were ovoid and large, with abundant cytoplasm and nucleated nuclei (Figure 3). Some lesions of dysplasia were noticed (Figure 3). The possible diagnoses included a seminomatous tumour with a cystic foci, a non seminomatous tumour with cystic foci, thymic carcinoma with a cystic degeneration, a mesothelial cyst degenerated into a mesothelioma, a bronchogenic cyst degenerated into an adenocarcinoma, and an oesophageal cyst degenerated into an adenocarcinoma. Immunohistochemical evaluation was performed using cytokeratin, CD30, PLAP, CD10, cytokeratin 7, cytokeratin 20, CD5, alpha-feto-protein, TTF1, WT1, calretinin, and mesothelin antibodies. The expression of cytokeratin antigen by the tumour cells was detected (Figure 3). The negativity of PLAP, alpha-feto-protein, and CD30 allowed ruling out the diagnoses of seminomatous or non seminomatous tumour. The negativity of WT1, claretinin, and mesothelin allowed ruling out a mesothelial nature of the cyst and mesothelial nature of the proliferation. The negativity of CD5 helped to rule out a thymic carcinoma. Negativity of CD10, cytokeratin 7, and cytokeratin 20 helped to rule out a possible metastasis. The negativity of TTF1 helped to rule out a pulmonary origin. The diagnosis of a degenerated oesophageal cyst was ruled out because an oesophageal cyst is lined by a multilayer lining. The final diagnosis was degeneration of a bronchogenic cyst.

**Figure 1:**
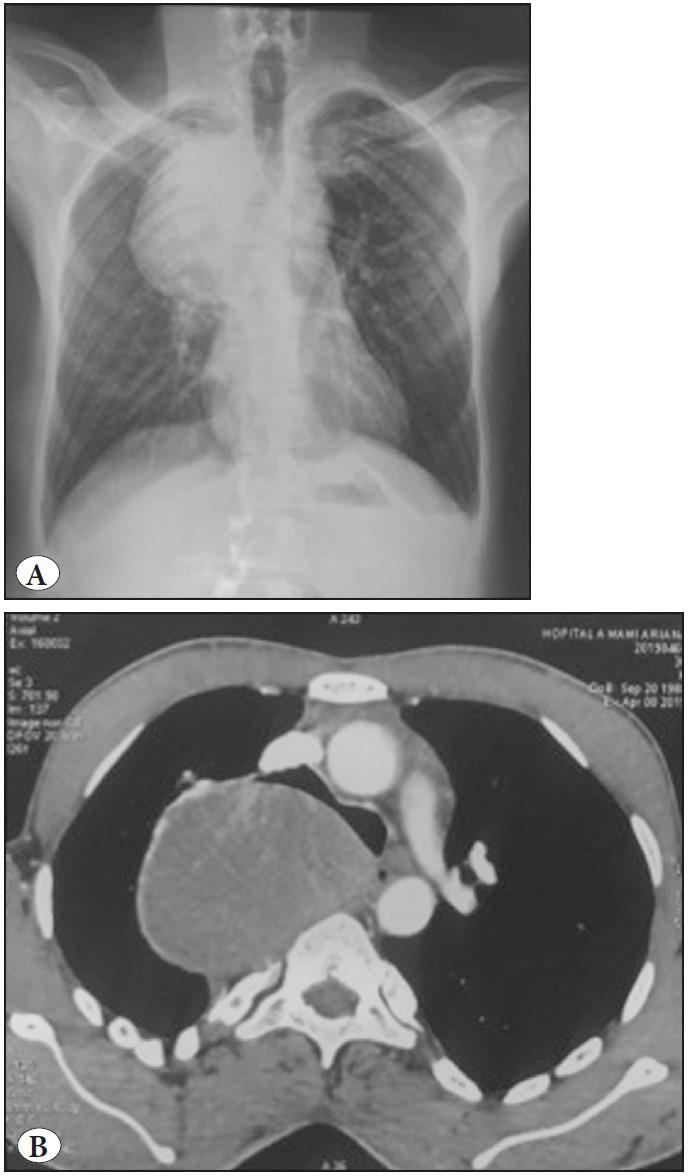
**A)** Chest-X-ray showing a 10 cm latero-tracheal shadow. **B)** CT-scan revealing a posterior and mediastinal mass in contact with D2 and D4 measuring 82x68 mm without costal invasion or lymph node metastases.

**Figure 2:**
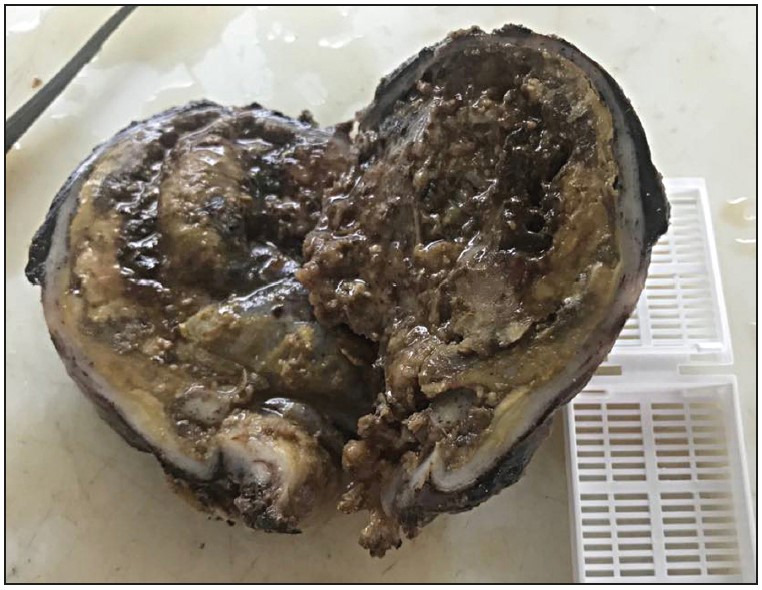
Gross features of a cystic mass with a necrotic content.

**Figure 3:**
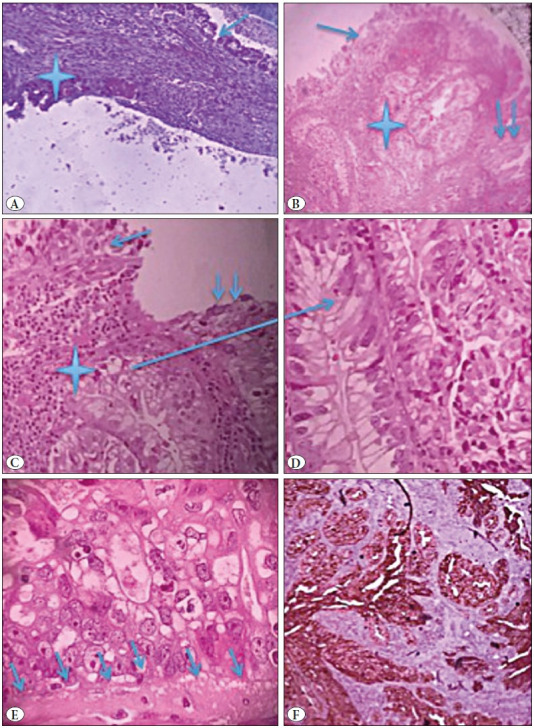
**A)** Microscopic features showing a fibrous cystic wall lined by a single-layer lining (arrow) with some calcifications (star) (H&E; x200). **B)** In some foci, the cystic wall is thickened and lined by a unistratified epithelium (arrow) and hyperplastic epithelium (double arrow) with carcinomatous foci (star) (H&E; x200). **C)** Carcinomatous foci (star) within a cystic wall with a unistratified layer (arrow) and a hyperplastic epithelium (double arrow) (H&E; x250). **D)** Carcinomatous foci made of large cells with abundant clear cytoplasm and nucleated nuclei (H&E; x400). **E)** Microscopic features showing dyplastic lesions within atypical cells limited by a preserved basement membrane (arrow) (H&E; x400). **F)** Immunohistochemical features showing the expression of the cytokeratin antibody by the tumour cells (IHC; x200).

The authors certify that appropriate patient consent was obtained.

Mediastinal cysts are benign lesions dominated by broncho-genic cysts in the anterior and middle mediastinum, mesothelial cysts in the middle mediastinum, and oesophageal cysts in the posterior mediastinum (1–3). They are mainly asymptomatic or have a nonspecific presentation (2). In the current case, the presentation was nonspecific and evoked mainly a diagnosis of tuberculosis. Imaging findings may be suggestive of the diagnosis when faced with a cystic lesion. The posterior location of the cyst makes the radiologists consider a neurogenic tumour, mainly a schwannoma or neurofibroma with cystic degeneration (4,5). A positive diagnosis is based on the microscopic exam. The characteristics of the lining are indicative of the diagnosis (6,7). Mesothelial cysts are lined by mesothelial cells that express mesothelial markers such as calretinin and sometimes epithelial markers consisting mainly of cytokeratin antibody. Oesophageal cysts have a gastric lining while bronchogenic cysts have a lining of respiratory epithelium together with bronchogenic glands and muscle fibres in the wall. In our case, the cystic lining was mainly ulcerated with rare remnant foci containing cylindrical or mesothelial-like cells. Besides, some muscle fibres were observed. Immunohistochemistry revealed the expression of epithelial markers by tumour cells, allowing them to show their epithelial nature. In this case, the nature of the cyst was challenging because of the predominance of degenerated foci in the form of adenocarcinoma.

The degeneration of mediastinal cysts has been rarely reported in the literature (5,8–12). This case was well illustrated because it represented a dysplastic lesion with a cystic lining showing a continuum with adenocarcinomatous foci.

## Conflict of INTEREST

The authors declare no conflict of interest.
